# Supplementary material for: DMRforPairs: identifying Differentially Methylated Regions between unique samples using array based methylation profiles
Source: BMC Bioinformatics. 2014 May 15;15:141. doi: 10.1186/1471-2105-15-141 (PMC4046028; doi:10.1186/1471-2105-15-141)
Supplement: Additional file 2 — DMRforPairs output for the comparison of A431-MCF7 and NA17018-NA17105. Please start from the HTML files in each folder. Available via the BMC Bioinformatics website. [file 1471-2105-15-141-S2.zip › 1394847754114233_MOESM2_ESM/A431_MCF7/figures/22654.pdf]

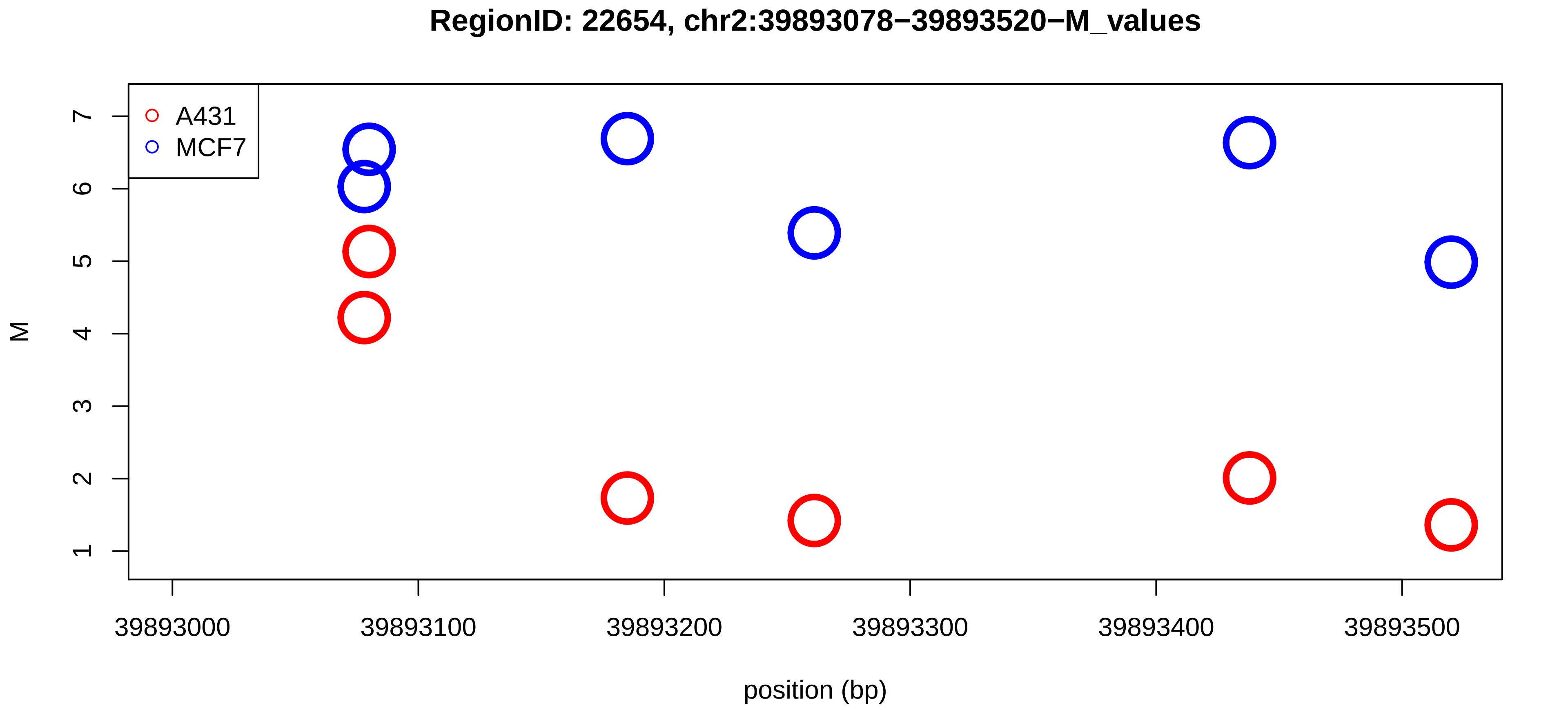

RegionID: 22654, chr2:39893078−39893520−M_values
A431
MCF7
M
1
2
3
4
5
6
7
39893000
39893100
39893200
39893300
39893400
39893500
position (bp)

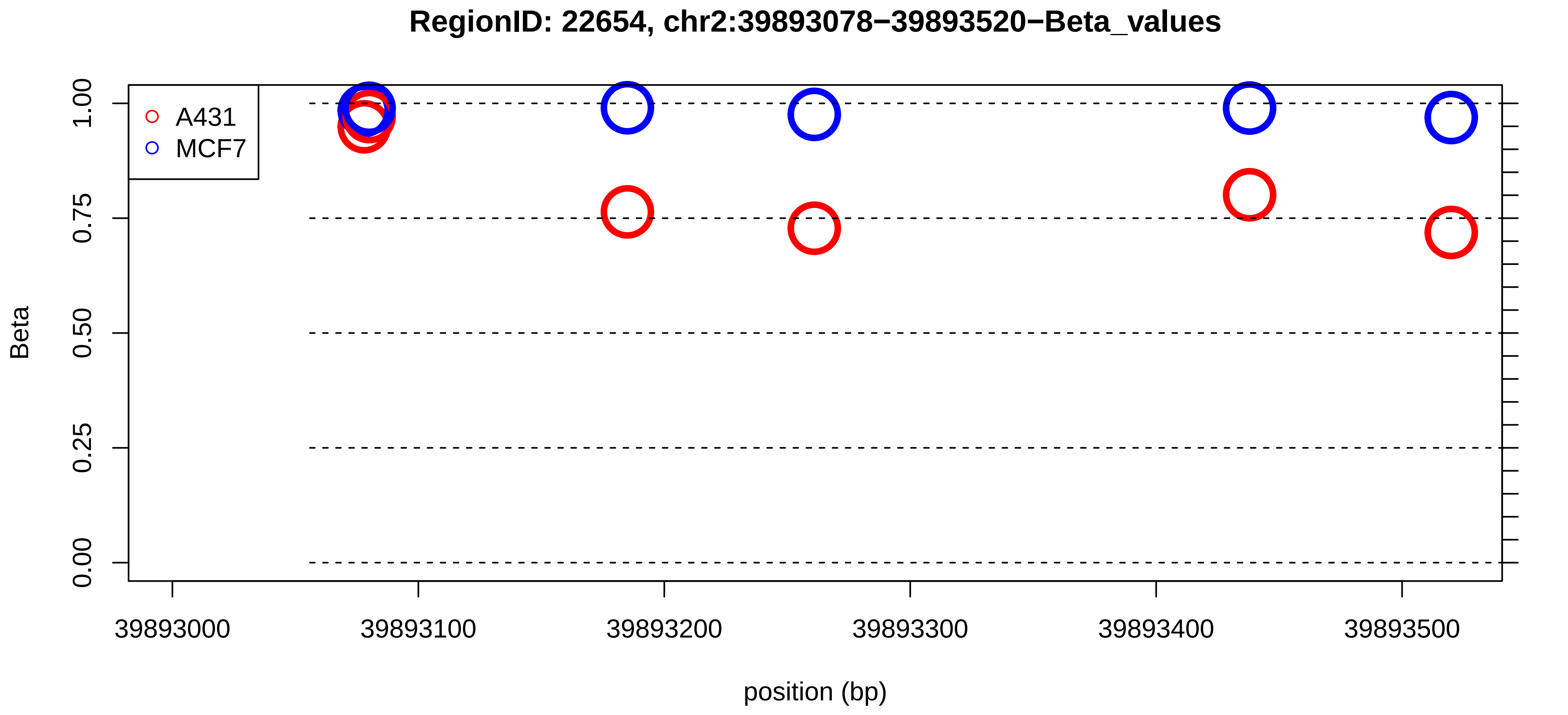

RegionID: 22654, chr2:39893078−39893520−Beta_values
A431
MCF7
Beta
0.00
0.25
0.50
0.75
1.00
39893000
39893100
39893200
39893300
39893400
39893500
position (bp)

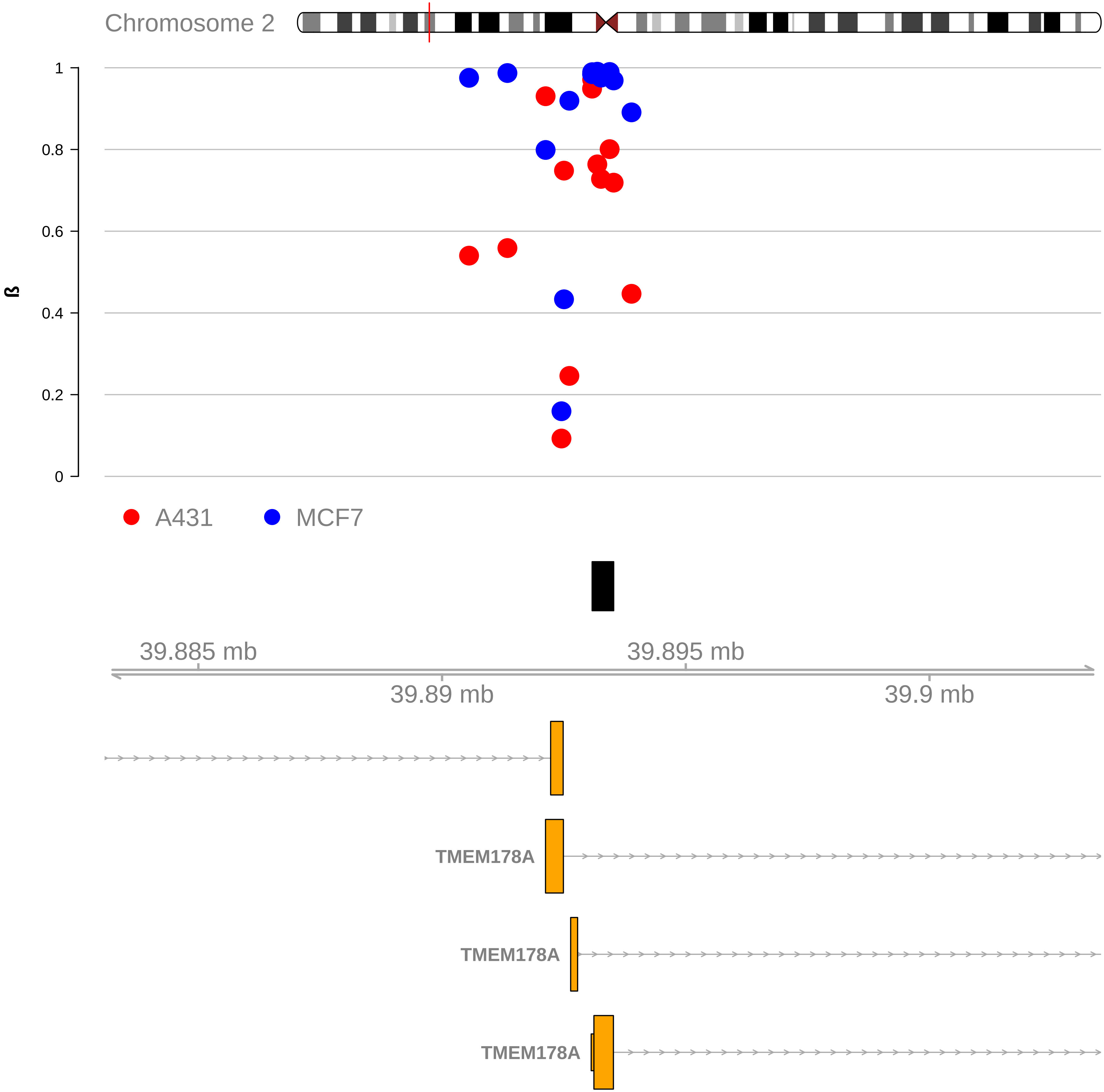
Chromosome 2
ß
1
0.8
0.6
0.4
0.2
0
A431
MCF7
39.885 mb
39.895 mb
39.89 mb
39.9 mb
TMEM178A
TMEM178A
TMEM178A
